# Social influence and consensus building: Introducing a q-voter model with weighted influence

**DOI:** 10.1371/journal.pone.0316889

**Published:** 2025-01-24

**Authors:** Pratik Mullick, Parongama Sen

**Affiliations:** 1 Department of Operations Research and Business Intelligence, Politechnika Wrocławska, Wrocław, Lower Silesia, Poland; 2 Department of Physics, University of Calcutta, Kolkata, West Bengal, India; AGH University of Krakow: Akademia Gorniczo-Hutnicza im Stanislawa Staszica w Krakowie, POLAND

## Abstract

We present a model of opinion formation where an individual’s opinion is influenced by interactions with a group of agents. The model introduces a novel bias mechanism that favors one opinion, a feature not previously explored. In the absence of bias, the system reduces to a mean field voter model. We identify three regimes: favoring negative opinions, favoring positive opinions, and a neutral case. In large systems, equilibrium outcomes become independent of group size, with only the bias influencing the final consensus. For smaller groups, however, the time to reach equilibrium depends on group size. Our results show that even a small initial bias leads to a consensus, with all agents eventually sharing the same opinion if the bias is not zero. The system also exhibits critical slowing down near the neutral bias, which acts as a dynamical threshold. The time to reach consensus scales logarithmically for non-neutral biases and linearly with system size for the neutral case. While short-term dynamics are influenced by group size, long-term behavior is determined solely by the bias.

## Introduction

Consensus formation [1–7] is a collaborative process that aims to achieve general agreement within a group, emphasizing open communication, mutual respect, and active participation. Unlike majority voting [8], consensus building integrates diverse perspectives, often through negotiation and compromise, leading to collective decision-making that could be essential in community planning [6], organizational environment [3], policy development [2] and social networks [5]. Collective decision-making [5, 7] in a social group is vital, as it harnesses diverse perspectives and leverages the group’s collective intelligence, leading to more informed, innovative, and effective solutions to complex problems. Modeling opinion dynamics helps us in studying consensus building [4, 9] or collective decision-making [10, 11], by analyzing social influence [12], peer interactions [13], and information dissemination [14]. Other factors such as the role of opinion leaders [9, 15], the impact of network structures [16], and the effect of external factors such as mass media [17, 18] are also taken into account to help us identify (i) the conditions under which consensus is more likely to be achieved, (ii) the mechanisms driving opinion convergence, and (iii) the potential barriers to collective decision-making.

The basic models of opinion dynamics [19, 20] provide frameworks for understanding how individual opinions evolve and aggregate within a social group, contributing to consensus building and collective decision-making. One of the earliest models, namely the voter model [21–23], has individuals randomly adopting a neighbor’s opinion, showing how majority opinions emerge over time. In the simplest case, the opinions are binary, i.e., assigned a value ±1. Subsequently, various models following the Sznajd model [24] have been proposed where several agents, as a group, influence the opinion of an agent. It is important to summarize some of these earlier works to emphasise how the model proposed in the present paper is different from them.

A nonlinear voter model was proposed by Castellano et. al. [25], where a selected agent interacts with *q* other neighbours. When the *q*-panel is unanimous, the agent selects their opinion—a situation known as conformity, otherwise the opinion is flipped with a probability. This model is also known as the *q*-voter model in literature. The dynamics are different from that of majority rule model [26, 27], where a *q*-panel is selected, and all the members belonging to the panel take the majority opinion within them. In [28], the agents are initially segmented into two groups—biased and unbiased—that remain fixed throughout the dynamics. The unbiased agents exhibit original voter dynamics, and the biased agents flip their opinions with probabilities that depend on their original own opinion and the opinion of their neighbour. Another interesting variant of the voter model is the noisy voter model where spontaneous flippings of the opinions are allowed [29].

A variant of the *q*-voter model was studied on complete graph [30] to understand social diffusion of innovation where the randomly chosen agent becomes either independent (not influenced by her *q* neighbours) or becomes conformist with the complementary probability. In [31], a *q*-voter model was studied on a complete graph with some ‘zealots’ in the system, who are inflexible with their opinions and do not change state under any conditions; with the susceptible agents maintaining conformist behaviour with a unanimous *q*-panel. Nyczka et. al. studied three different models in [32], each with conformist and one type of non-conformist agents, i.e., either anti-conformist (focal agent takes the opinion opposite to the one in unanimous *q*-panel) or independent agents. Another variant of *q*-voter model was studied in [33] where the agents are either conformist with a probability, or anti-conformist with the complementary probability; without considering any of them to be independent. A conformist *q*-voter model was also studied on a one dimensional lattice [34], where a consecutively indexed *q*-panel was chosen randomly. In case of unanimity in this *q* neighbourhood, either both the adjacent agents, or one of the 2 adjacent agents conforms to their opinion.

Variants of the *q*-voter model were also studied on multiplex networks [35], duplex clique [36], Erdos-Renyi graphs [37] and scale free networks [37]. A typical variant called the threshold *q*-voter model was studied in [38] on a complete graph, where unanimity within the *q*-panel is sufficient for a minimum number *q*_0_ of agents (0 ≤ *q*_0_ ≤ *q*) to influence the opinion of the focal agent, at the same time keeping the possibility of its independence. This threshold *q*-voter model was later studied on random networks in [37]. In [39] a generalized threshold *q*-voter model was studied with all basic type of social responses viz. conformity, anticonformity, independence, and uniformity/congruence. Recently, Muslim et. al. [18] studied a *q*-voter model where in case of non-unanimity in the *q*-panel the focal agent chooses an opinion expressed by the mass media with a probability. A similar model was studied in [17], where the independent agent might become skeptical of its own opinion, triggered by an unreliable external field in social processes, quite similar to how mass media influences the decision making in our society.

In this paper, we propose a *q*-voter model with binary opinions (say, positive or negative) where the independent behaviour of the focal agent is absent in general. Rather the *q*-panel, even in case of non-unanimity, influences the opinion of the agent under consideration, depending on the composition of the two opinions in this panel. Precisely, we consider that the agents have an influential power depending on their current opinion (positive or negative), with which they influence the opinion of the chosen agent. The weighted influential powers of both types of opinionated agents in the *q*-panel decide the opinion of the focal agent.

Rest of the paper is organised as follows. In the next section we define the dynamics of our model, followed by the discussion on our main results. We use mean field theory to find analytical solutions of our model, and also obtain results from Monte Carlo simulations to make comparisons with analytical expressions. In the final section we make some concluding remarks.

## Model description and features studied

In this model we consider a population of *L* agents with binary opinions on a complete graph. The opinions are either positive or negative. At each elementary step a randomly selected agent, say *A*, interacts with *q* ≥ 2 other agents, who are also chosen randomly. This set of *q* agents are named as the *q*-panel of *A*. If this *q*-panel is unanimous, *A* takes their opinion, a situation known as conformity. When the *q*-panel is not unanimous, we consider the *influential power* of agents in the *q*-panel. We assume that agents with positive and negative opinions have influential powers *p* and 1 − *p* respectively. In this model, since the *q* neighbors are randomly selected each time, the focal agent interacts with a different set of agents on each occasion. This scenario more closely resembles real life, where individuals are unlikely to engage in discussions with the same group of people every time.

In the *q*-panel, let there be *n* agents with positive opinion. These *n* agents with influential power *p* each, would try to convince *A* with a total influential power of *np*. On the other hand, *q* − *n* agents with negative opinion each with influential power 1 − *p*, would have a total influential power of (*q* − *n*)(1 − *p*).

Since *A* could take either the positive opinion or the negative opinion, the total probability of taking these two opinions at an elementary update should be 1. So if *p*_*q*+_ and *p*_*q*−_ denote the probabilities that *A* could take the positive opinion and the negative opinion respectively, then

$$\begin{array}{c} p_{q+}=\frac{np}{p(2n-q)+(q-n)} \end{array}$$ (1)

$$\begin{array}{c} p_{q-}=\frac{\left(q-n)(1-p\right)}{p(2n-q)+(q-n)}. \end{array}$$ (2)

**Fig 1:**
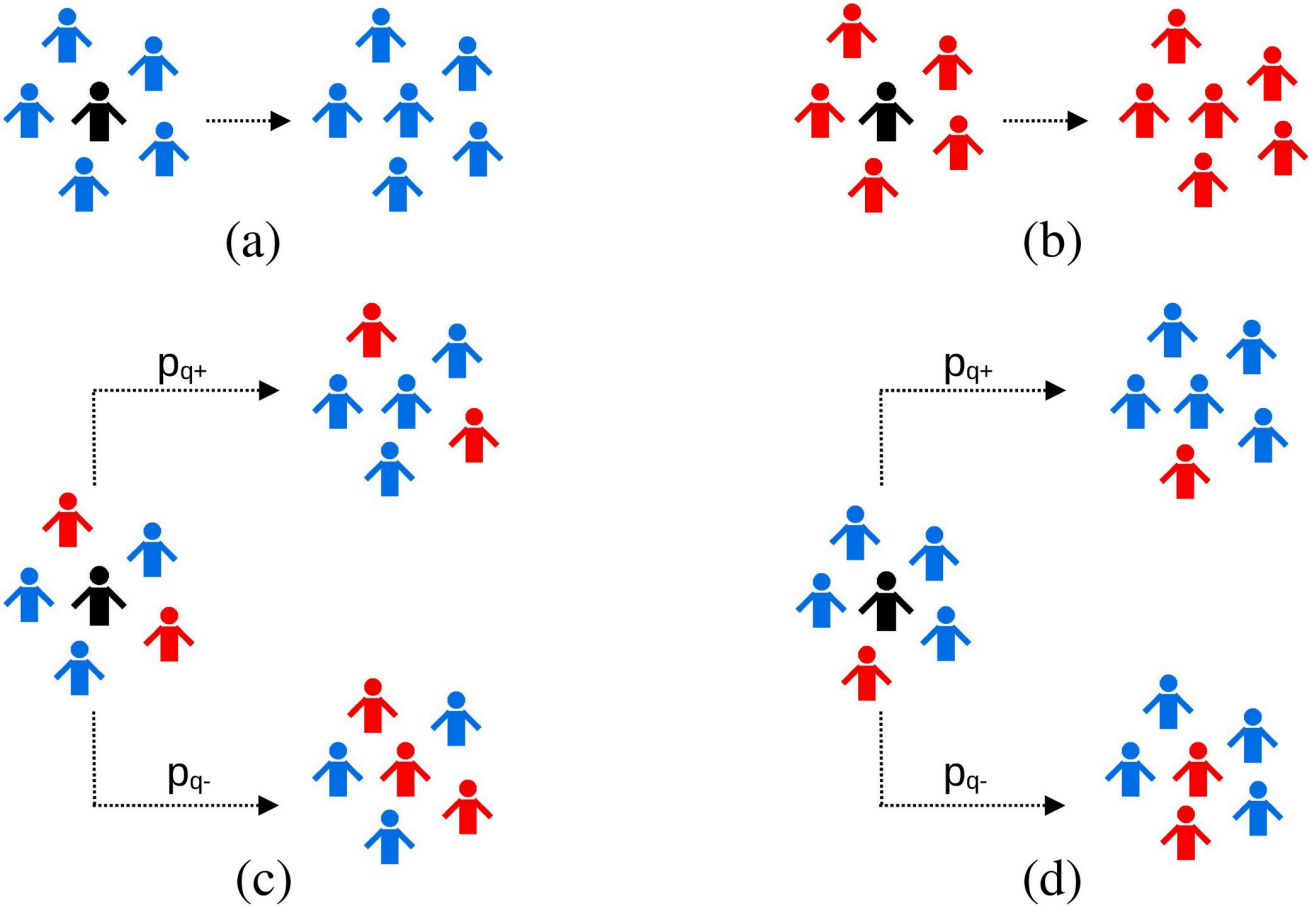
Schematic diagram showing the dynamics of our agent-based modelling. Here we show a typical case when a selected agent (shown in black) interacts with randomly chosen 5 other agents, i.e., *q* = 5. Case (a) shows conformity, when the *q*-panel of selected agent is comprised of all agents with positive opinion (shown in blue). Similarly, case (b) also represents conformity, the *q*-panel has only agents with negative opinion (shown in red). In case (c), the selected agent is surrounded by 3 agents with positive opinion and 2 agents with negative opinion, and in case (d) it is surrounded by 4 agents with positive opinion and 1 negative agent. In both these cases, the selected agent chooses positive opinion with probability *p*_*q*+_ and negative opinion with probability *p*_*q*−_. The expressions for *p*_*q*+_ and *p*_*q*−_ are given by Eqs (1) and (2) respectively.

*p*_*q*+_ and *p*_*q*−_ could be seen as the weighted average of influential powers of *n* agents with positive opinion and *q* − *n* agents with negative opinion. The term *np* + (*q* − *n*)(1 − *p*) = *p*(2*n* − *q*) + (*q* − *n*) is the normalisation factor. The dynamics of the model are schematically shown in Fig 1. It can be easily checked from Eqs (1) and (2) that if *n* = 0 or *q*, there is conformity. For *n* = *q*/2 (assuming *q* is even), *p*_*q*+_ = *p*, clearly indicating the bias for *p* ≠ 0 even when agents with the two opinions are present in equal numbers.

For *q* = 2 and *p* = 1/2 the dynamics are identical to that of a *q*-voter model with *q* = 2 [25]. For *p* = 1/2, for any value of *q* when all the agents are equally influential, *p*_*q*+_ = *n*/*q*. This is equivalent to a mean field voter model [21] with *q* neighbours (the *q* neighbours vary, therefore it is mean field like) in which an agent picks up any random neighbour such that the probability to adopt the positive opinion is *n*/*q*. When the *q*-panel is unanimous, all the neighbours have the same state so the choice is unique.

Let *f*_+_(*t*) be the fraction of agents with positive opinion in the whole system at any time *t*. The initial fraction of agents with positive opinion is denoted by *x* = *f*_+_(0) throughout this paper. Obviously, *f*_−_(*t*), the fraction of agents with negative opinions is equal to 1 − *f*_+_(*t*). We use mean field theory to obtain the dynamical equations governing *f*_+_(*t*) and solve the equations. We also make a fixed point analysis and a linear stability analysis for small *q* values as well as for *q* → ∞. Also, using Monte Carlo simulations, we study *f*_+_(*t*) as a function of time and compare with the mean field results. In general, we find that the final state is a consensus state with all the opinions becoming either positive or negative. An estimate of time scales has been made from both the mean field theory and numerical simulations.

We employ numerical simulations to estimate the exit probability *E*(*x*), a quantity of interest in opinion dynamics models. This is defined as the probability that the system would reach a positive consensus state after starting from an initial fraction *x* of agents with positive opinion. In particular, for the voter model, the exit probability follows a linear behaviour *E*(*x*) = *x*, which is a consequence of the conservation of the fraction of agents with either opinion in the ensemble. We present also the results for the time taken to reach the consensus state as a function of the system size in the simulations. All quantitative studies are performed by varying the two independent parameters *q* and *p*.

## Results and discussion

### Mean field approach

We consider a fully connected network of *L* agents and hence the mean field theory can be used here. Let time be updated as *t* → *t* + 1/*L* and at each time step, the fraction of agents having any type of opinion can increase/decrease by 1/*L*. One can then write down the master equation for *f*_+_(*t*), the probability that an agent has a positive opinion, involving the transition rates for the two states.

For the present model, with *L* → ∞, the transition rates *ω* between a positive (+) state and a negative (−) state and vice versa are given by

$$\begin{array}{c} \omega_{-\to +}=f_{+}^{q}+\sum_{n=1}^{q-1}p_{q+}\left(\begin{array}{c} q\\ n \end{array}\right){f_{+}}^{n}{\left(1-f_{+}\right)}^{q-n} \end{array}$$

$$\begin{array}{c} \omega_{+\to -}={\left(1-f_{+}\right)}^{q}+\sum_{n=1}^{q-1}p_{q-}\left(\begin{array}{c} q\\ n \end{array}\right){f_{+}}^{n}{\left(1-f_{+}\right)}^{q-n}, \end{array}$$

where in the RHS, the first terms correspond to conformity, i.e. when all the *q* agents have the same opinion (positive and negative for the two equations respectively) and the second terms include all other cases. The master equation for *f*_+_ can be written as

$$\begin{array}{c} \frac{df_{+}}{dt}=-\omega_{+\to -}f_{+}\left(t\right)+\omega_{-\to +}f_{-}\left(t\right), \end{array}$$ (3)

such that one gets on simplification

$$\begin{array}{c} \frac{df_{+}}{dt}=\sum_{n=0}^{q}p_{q+}\left(\begin{array}{c} q\\ n \end{array}\right){f_{+}}^{n}{\left(1-f_{+}\right)}^{q-n}-f_{+}. \end{array}$$ (4)

Note that in Eq 4, if one puts *p* = 1/2 then with *p*_*q*+_ = *n*/*q*, we get $\frac{df_{+}}{dt}=0$ irrespective of the value of *q*, as the system behaves as a mean field voter model with *q* neighbours; any value of *f*+ is a fixed point here. Also, the equation is consistent with the fact that *f*_+_ = 0, 1 are trivial fixed points.

The first term on the RHS of Eq 4 involves a sum which becomes difficult to handle for larger values of *q*. However, for small values of *q*, one can evaulate the terms to see how the system evolves. In the following, we discuss the cases for *q* = 2 and 3.

#### *q* = 2 case

The master Eq 14 for *q* = 2 reduces to

$$\begin{array}{c} \frac{df_{+}}{dt}=f_{+}\left(1-f_{+}\right)\left(2p-1\right) \end{array}$$ (5)

which shows that there are only two fixed points for any *p* ≠ 0.5 at *f*_+_ = 0, 1.

Let us now consider a model, which we call the binary model with stochastic biased flipping or BMSBF. Here, only pairwise interactions are allowed and if an agent with positive opinion interacts with one with a negative opinion, her opinion flips with probability (1 − *p*) (compare this with the voter model where this occurs with probability 1). Similarly, for an agent with negative opinion, her opinion flips with probability *p*. So *p* = 1/2 here is a case of unbiased flipping, which implies that flippings occur with probability 1/2 whenever one interacts with an agent with the opposite opinion. In this model, one can formulate the mean field master equation as

$$\begin{array}{c} \frac{df_{+}}{dt}=f_{+}f_{-}p-f_{+}f_{-}\left(1-p\right). \end{array}$$ (6)

Interestingly, this coincides with Eq (5) such that one can interpret the *q* = 2 model for any *p* as a BMSBF. The purpose of showing the equivalence of the *q* = 2 model to a distinctly non-Voter like model for general values of *p* ≠ 1/2 is to emphasize that the present model differs from the voter model in general.

The solution of Eq (5) can easily be obtained as

$$\begin{array}{c} f_{+}=\frac{Ae^{(2p-1)t}}{1+Ae^{(2p-1)t}}, \end{array}$$ (7)

where $A=\frac{f_{+}\left(0\right)}{1-f_{+}\left(0\right)}$.

Eq (5) shows that there are two fixed points $f_{+}=f_{+}^{*}=0,1$ for any *p*. Let *δ* be defined as the infinitesimal deviation from a fixed point. Putting $f_{+}=f_{+}^{*}+\delta$, where *δ* is negative for $f_{+}^{*}=1$, one gets up to linear order in *δ* (for small values of |*δ*|),

$$\begin{array}{c} \frac{d\delta }{dt}=\delta \left(1-f_{+}^{*}\right)\left(2p-1\right)-\delta f_{+}^{*}\left(2p-1\right). \end{array}$$ (8)

This leads to an exponential time dependence of *δ* as follows:

$$\begin{array}{c} \left|\delta \right|\propto e^{\pm (2p-1)t} \end{array}$$ (9)

where the + (−) sign is for the fixed point $f_{+}^{*}=0$ ($f_{+}^{*}=1$). This shows that $f_{+}^{*}=0$ is unstable (stable) for *p* > 0.5 (*p* < 0.5) as *δ* grows (decreases), and for $f_{+}^{*}=1$, it is the opposite. This implies that whenever the initial configuration is biased towards the positive (negative) opinion, the final outcome would be a positive (negative) consensus for *p* > 0.5 (*p* < 0.5). This will be reflected in the behaviour of the exit probability to be discussed later.

We also note from Eq 9 that the exponents governing the time evolution are same in magnitude for both the fixed points. These exponents signify the (inverse of the) characteristic time scale with which infinitesimally close trajectories are separated or come closer in time *t*.

#### *q* = 3 case

The rate equation for *f*_+_ for this case is given by

$$\begin{array}{c} \frac{df_{+}}{dt}=\frac{6p}{p+1}f_{+}^{2}\left(1-f_{+}\right)+\frac{3p}{2-p}f_{+}{\left(1-f_{+}\right)}^{2}+f_{+}^{3}-f_{+}. \end{array}$$ (10)

To perform a linear stability analysis, as we did for the previous case, we put $f_{+}=f_{+}^{*}+\delta$ in Eq (10) and ignoring higher order terms in *δ* we get

$$\begin{array}{cc} \frac{d\delta }{dt} & =\frac{6p}{p+1}[2f_{+}^{*}\delta +\left(1-3\delta \right){f_{+}^{*}}^{2}-{f_{+}^{*}}^{3}]\\ & +\frac{3p}{2-p}[\left(1-4\delta \right)f_{+}^{*}+\left(3\delta -2\right){f_{+}^{*}}^{2}+{f_{+}^{*}}^{3}+\delta ]+{f_{+}^{*}}^{3}+3\delta {f_{+}^{*}}^{3}-f_{+}^{*}-\delta \end{array}$$ (11)

such that for $f_{+}^{*}=0$,

$$\begin{array}{c} \delta \propto e^{\frac{4p-2}{2-p}t} \end{array}$$ (12)

and for $f_{+}^{*}=1$ (for which *δ* is negative),

$$\begin{array}{c} \left|\delta \right|\propto e^{-\frac{4p-2}{1+p}t}. \end{array}$$ (13)

Hence although the stability behavior of the fixed points are similar, the timescales depend on the exact fixed points in contrast to the *q* = 2 case.

#### Larger *q* values: Simplified mean field theory

So far we considered mean field theory assuming *L* → ∞ but *q* finite. However, as the first term in Eq 4 becomes cumbersome for large *q* (even when handled numerically), we make an additional approximation and replace *n* by its average value which is *qf*_+_, in the sum in Eq 4. We call this the simplified mean field theory (SMFT) for convenience. This approximation is correct for *q* → ∞, however, we can still use it for finite *q* and check how it affects the result by comparing with the results of Monte Carlo simulations to be discussed later.

Using *n* = *qf*_+_ in Eqs (1) and (2), the transition rates *ω* between a positive (+) state and a negative (−) state are obtained as

$$\begin{array}{c} \omega_{-\to +}=f_{+}^{q}+\left[1-f_{+}^{q}-{\left(1-f_{+}\right)}^{q}\right]\times \frac{pf_{+}}{\left(1-f_{+}\right)\left(1-p\right)+f_{+}p} \end{array}$$

$$\begin{array}{c} \omega_{+\to -}={\left(1-f_{+}\right)}^{q}+\left[1-f_{+}^{q}-{\left(1-f_{+}\right)}^{q}\right]\times \frac{\left(1-p\right)\left(1-f_{+}\right)}{\left(1-f_{+}\right)\left(1-p\right)+f_{+}p}. \end{array}$$

Now the rate equation simplifies to

$$\begin{array}{c} \frac{df_{+}}{dt}=f_{+}^{q}\left(1-f_{+}\right)-{\left(1-f_{+}\right)}^{q}f_{+}+\left(1-f_{+}\right)f_{+}\frac{\left\{1-f_{+}^{q}-{\left(1-f_{+}\right)}^{q}\right\}\left(2p-1\right)}{\left(1-p\right)\left(1-f_{+}\right)+pf_{+}}. \end{array}$$ (14)

It maybe noted that there are two fixed points $f_{+}^{*}=0,1$ for all values of *p*, *q* and also a third, which is obtained after numerically solving the equation. The third fixed point in general, depends on both *p* and *q*. Also, if one puts *f*_+_ = *f*_−_ = 0.5 and *p* = 0.5 in the above equation, one gets $\frac{df_{+}}{dt}=0$, which implies that for any *q* this is the third fixed point.

For *q* → ∞ limit, the Eq (14) becomes

$$\begin{array}{c} \frac{df_{+}}{dt}=\left(1-f_{+}\right)f_{+}\frac{\left(2p-1\right)}{\left(1-p\right)\left(1-f_{+}\right)+pf_{+}} \end{array}$$ (15)

such that the fixed points are again simply $f_{+}^{*}=0,1$ and for *p* = 0.5, all points are fixed points.

We perform a linear stability analysis by substituting $f_{+}=f_{+}^{*}+\delta$ in Eq (15) to get

$$\begin{array}{c} \frac{d\delta }{dt}=\delta \frac{\left[1-2f_{+}^{*}\right]\left(2p-1\right)}{\left(1-p\right)\left(1-f_{+}^{*}\right)+pf_{+}^{*}}+O\left(\delta^{2}\right) \end{array}$$ (16)

Thus, for $f_{+}^{*}=0$ we get

$$\begin{array}{c} \frac{d\delta }{dt}=\frac{2p-1}{1-p}\delta . \end{array}$$ (17)

This indicates growth of *δ* for *p* > 0.5. Similarly for $f_{+}^{*}=1$ we get,

$$\begin{array}{c} \frac{d\delta }{dt}=-\frac{2p-1}{p}\delta , \end{array}$$ (18)

which indicates growth of *f*_+_(*t*) for *p* < 0.5. Since *δ* can not exceed 1 or be less than −1 for the two regions where we find growth (decay) of *f*_+_(*t*), we need vanishing contribution from *δ*, i.e., *f*_+_ = 1 + *δ*, where *δ* < 0 (say for $f_{+}^{*}=1$). Then using Eq (17) we get,

$$\begin{array}{c} f_{+}=1-|\delta |=1-|\delta_{0}|e^{-(\frac{2p-1}{p})t} \end{array}$$ (19)

Similarly, for $f_{+}^{*}=0$, say *f*_+_ = *δ* and $\delta =\delta_{0}e^{-(\frac{2p-1}{1-p})t},$ using Eq (18). This vanishes for *p* < 0.5. So in this region we have

$$\begin{array}{c} f_{+}=\delta_{0}e^{(\frac{2p-1}{1-p})t}. \end{array}$$ (20)

Although strictly speaking the SMFT is correct for large *q* values one can still put *q* = 2 or 3 in Eq (14) which leads to a third fixed point as mentioned earlier (e.g, for *q* = 2, the third fixed point is at (1 − *p*) for *p* ≠ 0.5). However, this is not true according to the general mean field calculations done without assuming *n* = *qf*_+_. Apparently the third fixed point is an artefact of this assumption for finite values of *q*. We will get back to this point later.

### Monte Carlo simulations

The mean field theory is exact for fully connected models, however, the effect of making the additional approximation of replacing *n* by a single averaged value in the SMFT may be significant for finite but large *q* values as discussed in the previous section. Hence we perform Monte Carlo simulations (see S1 File) of the model and compare the results with those found using the mean field approach.

The simulation begins *x* = *f*_+_(*t* = 0) fraction of agents with positive opinions in a system comprising *L* agents (i.e., the system size is *L*). For our scheme of simulation we use random asynchronous update, which means that at each time step one agent is randomly chosen and instantly updated according to the defined dynamical rule. *L* such updates are equivalent to one Monte Carlo (MC) step. Let us summarise the dynamical rule for our model as follows

1. Randomly select an agent *i* from 1 to *L*.
2. Randomly select *q* other agents such that none of these are the same as *i*. This was done using Fisher-Yates shuffle algorithm [40]. These *q* agents are selected without repetition, and termed as the *q*-panel with which agent *i* interacts.
3. If all of these *q* agents have the same opinion, i.e., the *q*-panel is unanimous, then agent *i* takes this opinion—a situation defined as conformity.
4. Otherwise, if the *q*-panel is non-unanimous, then we count the number of agents with positive opinion *n* in this panel, and then let agent *i* take the positive opinion with probability *p*_*q*+_ and the negative opinion with probability *p*_*q*−_. The expressions for *p*_*q*+_ and *p*_*q*−_ are given by Eqs (1) and (2).
5. Steps 1 to 4 are repeated *L* times. This constitutes one MC time step.
6. The simulations are then continued until a maximum number of MC steps, or until a global consensus is reached, i.e., each of *L* agents in the system has the same opinion.
7. Finally the results are averaged over several initial configurations.

**Fig 2:**
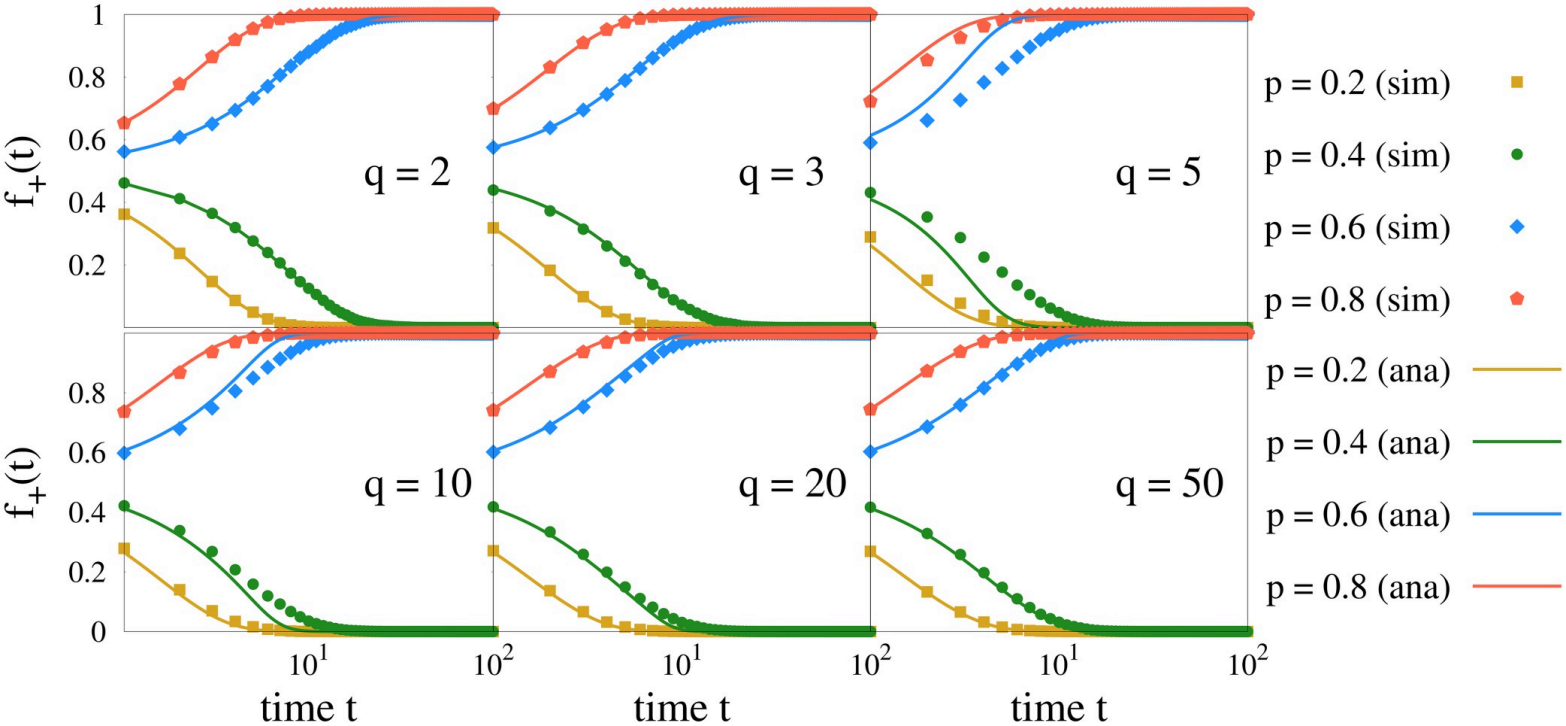
Variation of fraction *f*_+_ of agents with positive opinion as a function of time *t*. Plots are for different values of *p* & *q*, and *x* = 0.51. Simulated (sim) results are shown by solid circles, and analytical (ana) results are shown by solid lines. Simulations are performed using *L* = 1024 averaging over 100 configurations. The agreement between simulated and analytical results are excellent for *q* = 2 and 3. For cases with *q* ≥ 4 the agreement becomes more reasonable as *q* increases.

**Fig 3:**
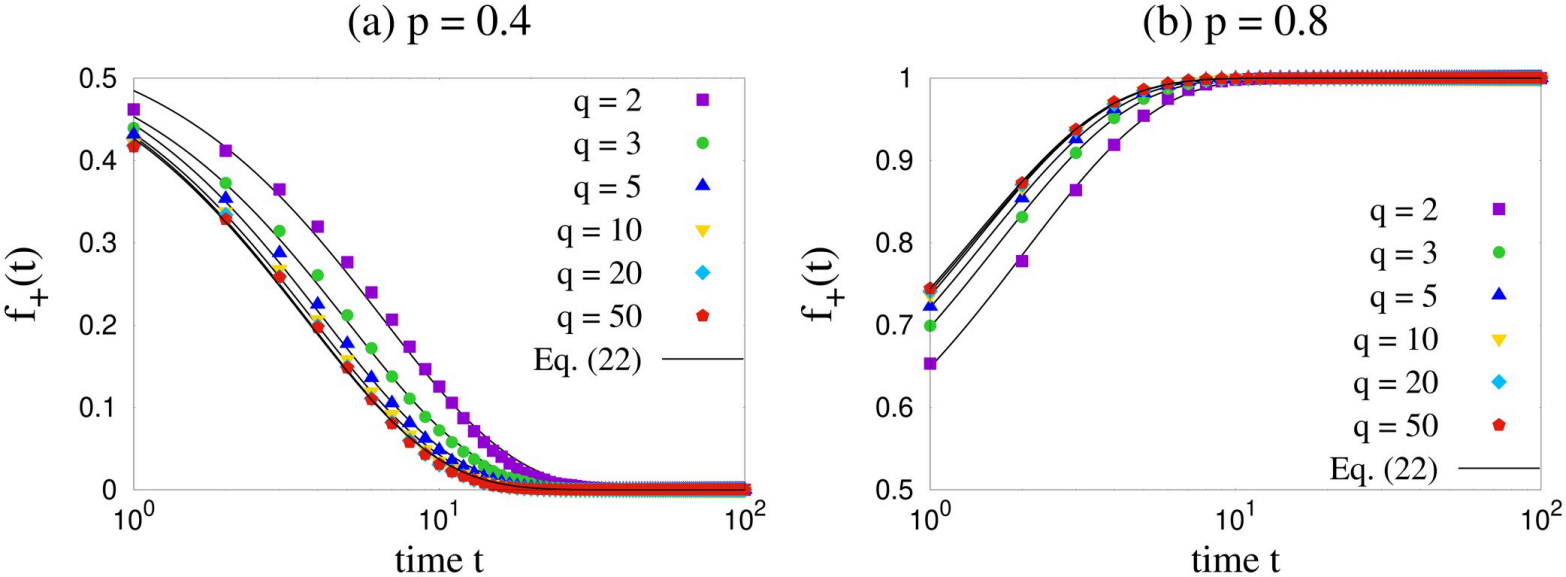
Fraction *f*_+_ of agents with positive opinion as a function of time *t*. The plots are for *x* = 0.51 for several values of *q* and two typical values of *p*, viz. (a) *p* = 0.4 and (b) 0.8. Simulations are done for *L* = 1024 on a complete graph. As *q* increases, *f*_+_ becomes *q* independent. The black curves are data fittings done using Eq (22).

First we calculate the fraction *f*_+_(*t*) of up spins as a function of time *t*. The results are shown in Figs 2 and 3. To compare the numerically simulated results with our analytical expressions, we use Eq (7) for the case *q* = 2, as it gives an exact solution for *f*_+_(*t*). However, we use Euler’s method to solve the differential equations given by Eq (10) for *q* = 3 and Eq (14) for *q* ≥ 4. Overall the results match qualitatively. We observe that for *q* = 2 and 3, numerical and analytical results agree in an excellent manner. For finite *q* values ≥ 4, there is a quantitative discrepancy which vanishes as *q* is made larger. Obviously, this discrepancy arises from neglecting the fluctuations in *q* in the simplified mean field theory. In the large *q* limit (e.g. *q* = 50) the agreement becomes excellent.

The absorbing states are the consensus states with *f*_+_ equal to zero or 1 (except for *p* = 0.5) as found from both the methods. The data presented in Fig 2 indicate that for *p* < 0.5 the fraction of agents with positive opinion becomes 0, and for *p* > 0.5 this fraction becomes 1 for the particular initial condition taken. To analyse further we varied the initial condition and calculated the exit probability *E*(*x*). This study helps in understanding the fate of the system depending on the initial condition and also enables us to obtain a finite size scaling form for *E*(*x*) and the related exponent, if any.

### Exit probability

**Fig 4:**
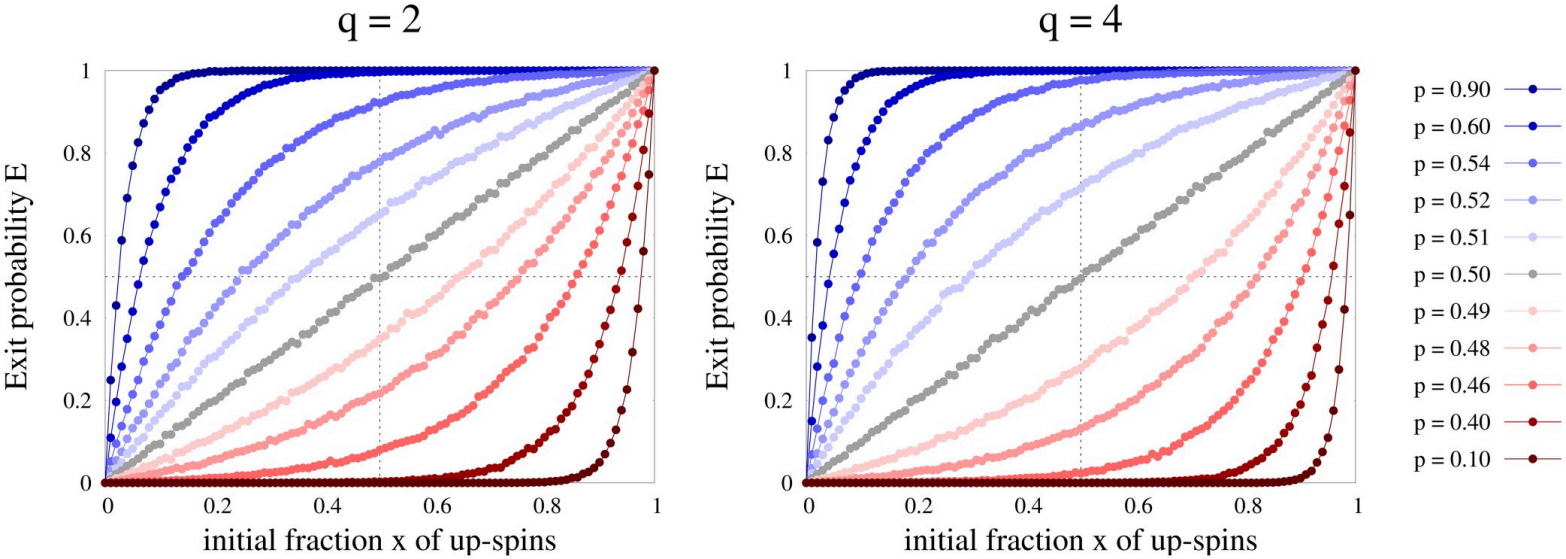
Exit probability *E*(*x*) as a function of initial fraction *x* of agents with positive opinion. Plots are shown for several values of *p* and two values of *q*. Simulation were done for *L* = 64 on a complete graph. The results are qualitatively similar across the values of *q* shown here.

**Fig 5:**
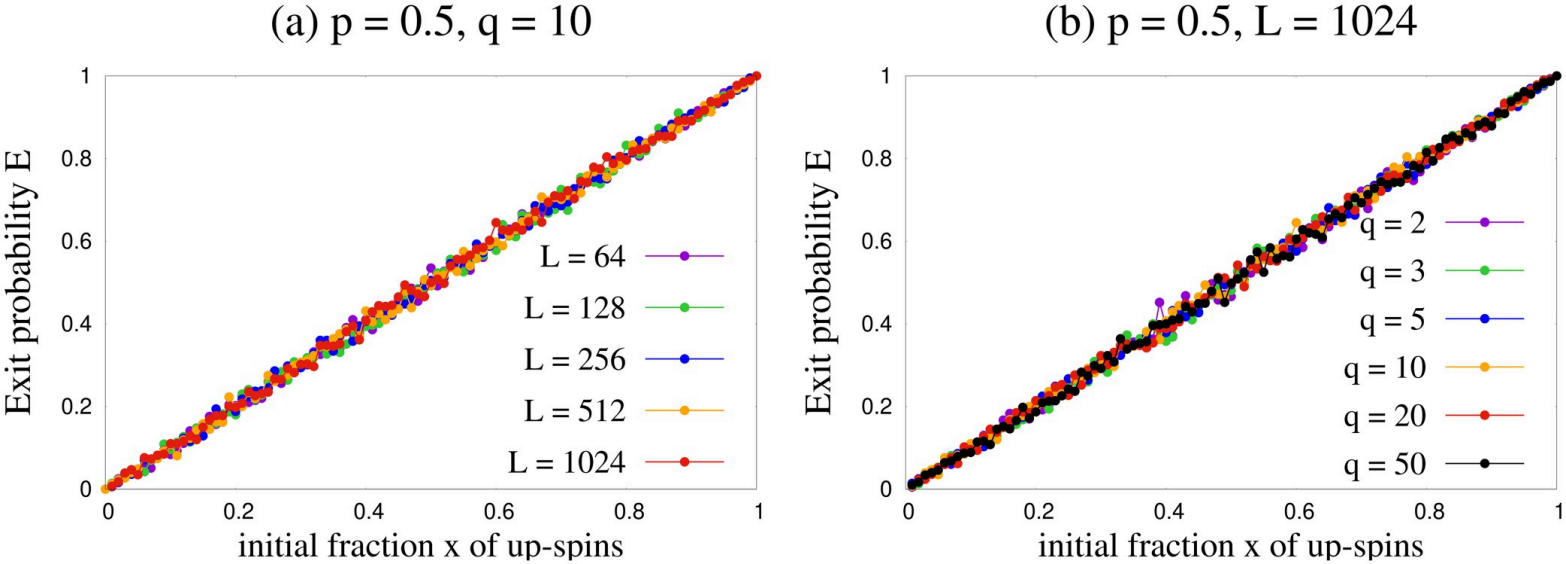
Variation of exit probability *E*(*x*) as a function of initial fraction *x* of up spins. Plots are shown for (a) several system sizes with *p* = 0.5, *q* = 10 and for (b) various values of *q* with *p* = 0.5, *L* = 1024. It seems that *E*(*x*) maintains its linear behaviour even in the thermodynamic limit and in large *q* limit.

Our results for exit probability using Monte Carlo simulations are shown for two values of *q* in Fig 4. Apparently, the qualitative behavior is independent of *q* when we study *E*(*x*) for lower values of *q*. Before discussing further on the dependence of the exit probability results on *q*, let us first focus on the point *p* = 0.5. From Fig 4 we can see that *E*(*x*) = *x* for *p* = 0.5 for all the values of *q* ≤ 5. Fig 5 shows the results for *p* = 0.5 for several values of *L* and *q*, and they are consistent with *E*(*x*) = *x*. This is compatible with our previous observation that for *p* = 0.5, the probabilities given by Eqs 1 and 2 become equivalent to a voter model.

**Fig 6:**
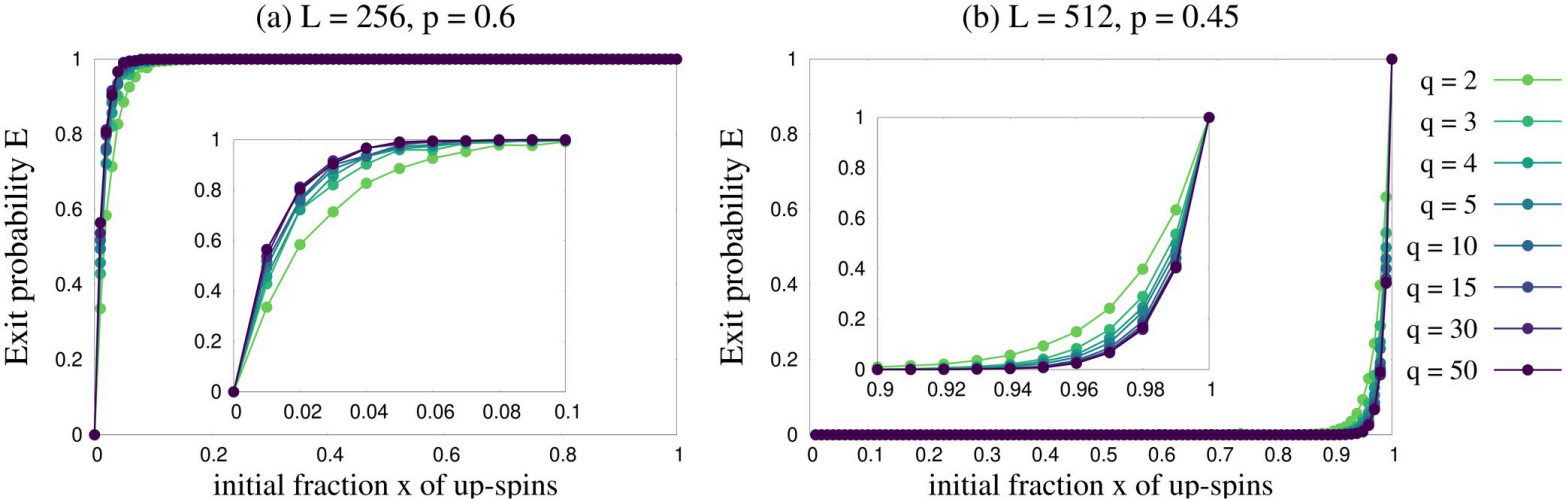
Exit probability *E*(*x*) as a function of initial fraction *x* of agents with positive opinion. Plots are shown for several values of *q* from 2 to 50 and for (a) *L* = 256, *p* = 0.6 and (b) *L* = 512, *p* = 0.45. The results converge as *q* grows larger.

Next we focus on the dependence of exit probability results on *q* for general *p* values. As already mentioned, for lower values of *q* the exit probability curves are qualitatively *q*-independent, as seen from Fig 4. To dig into this further, we numerically obtain *E*(*x*) versus *x* curves up to *q* = 50, and show their comparison with lower values of *q* in Fig 6. The exit probability curves actually converge as *q* takes larger values. This means that the steady states in our model do not depend on *q* as the value of *q* increases. This was also confirmed when we studied the trajectories for fraction *f*_+_ of agents with positive opinion (Fig 3).

**Fig 7:**
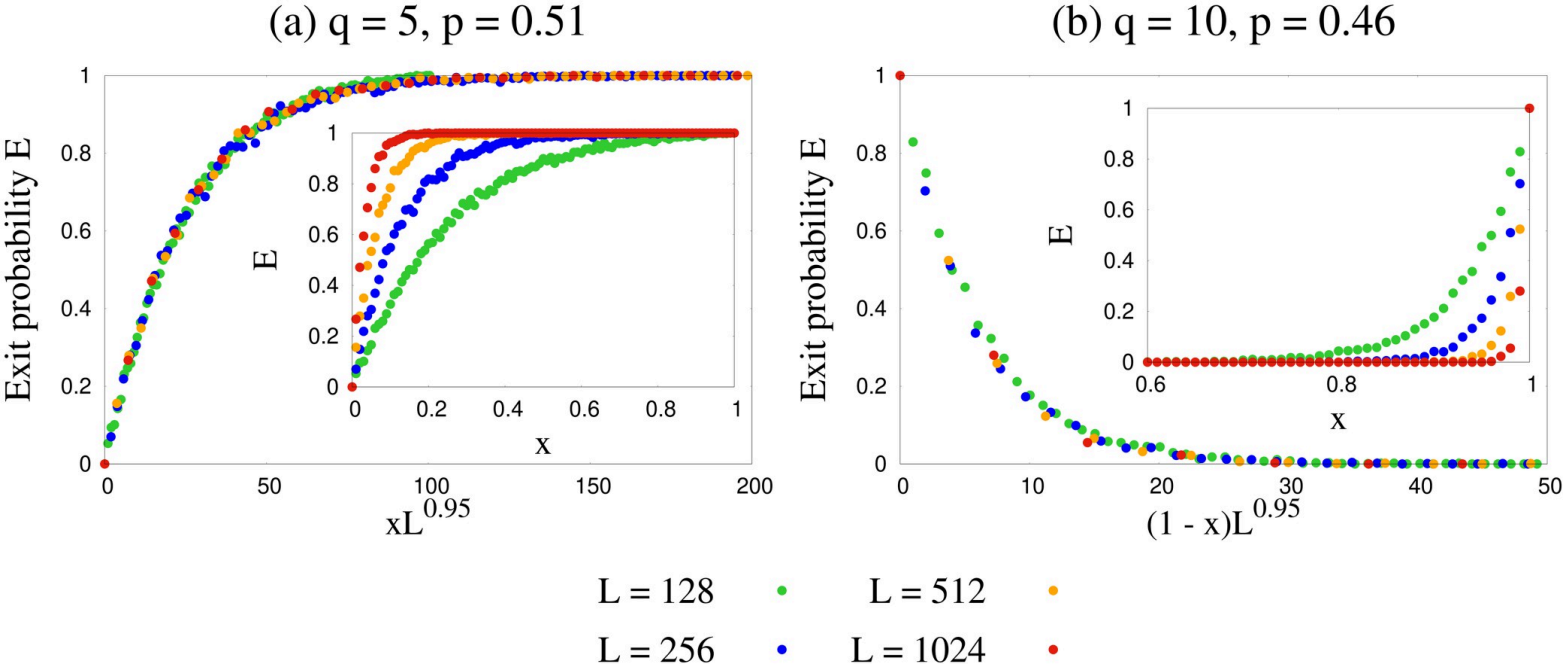
Data collapse of exit probability *E*(*x*). Plots are shown for several values of system size *L* from 128 to 1024 for (a) *q* = 5, *p* = 0.51 and (b) *q* = 10, *p* = 0.46. For *p* > 0.5 the scaling argument is *xL*^0.95^ and for *p* < 0.5 it is (1 − *x*)*L*^0.95^. Insets show the unscaled data. It is evident that in the thermodynamic limit *L* → ∞ the exit probability would become a step functions at *x* = 0 for *p* > 0.5 and at *x* = 1 for *p* < 0.5.

But how do our results depend on the system size *L*? To investigate the finite size effect of exit probability we simulated our model for several system sizes from *L* = 64 to 1024 and have shown the results in the insets of Fig 7. It is clear that in the thermodynamic limit *L* → ∞ the exit probability exhibits a step function like behavior even for a minor deviation from *p* = 0.5. We can see from Fig 7 that for *p* > 0.5 the exit probability would show a step function at *x* = 0, and for *p* < 0.5 it would show a step function at *x* = 1 in thermodynamic limit. The implication of this observation is very critical from the perspective of opinion dynamics in human societies. It indicates that even if in the beginning we have a very small fraction *x* of agents with positive opinion, the system could still reach a positive consensus given the influential power *p* of agents with positive opinion is slightly higher than that of agents with negative opinion. Quite similarly, if initially we have a very large fraction *x* of agents with positive opinion, the system could still reach a negative consensus given the influential power *p* of agents with positive opinion is slightly smaller than that of agents with negative opinion. The parameter *p*, the influential power of agents with positive opinion, thus introduces a broken symmetry in the dynamics, irrespective of the value of *q*. The data obtained for the exit probability *E*(*x*) were collapsed using the following scaling forms:

$$\begin{array}{cc} E(x) & =g_{1}\left(xL^{\nu }\right)\;\text{for}\;p>0.5\\ & =g_{2}\left(\left(1-x\right)L^{\nu }\right)\;\text{for}\;p<0.5, \end{array}$$ (21)

where we found a universal value of *ν* ≃ 0.95 for any *q*. The collapsed data was found to fit well according to the functional form *g*_1_(*z*) = 1 − exp(−*z*/*b*) for *p* > 0.5 and *g*_2_(*z*) = exp(−*z*/*b*′) for *p* < 0.5, where the parameters *b* and *b*′ become *q*-independent as *q* increases. The form of the scaling functions indicate that *b* and *b*′ are like scales governing the approach to unity for *p* > 0.5 or to zero for *p* < 0.5 respectively for the exit probability. The saturated values of 1/*b* and 1/*b*′ show *p*-dependence, much similar to *β* and *β*′ (see Eqs 23 and 24 in the next section).

**Fig 8:**
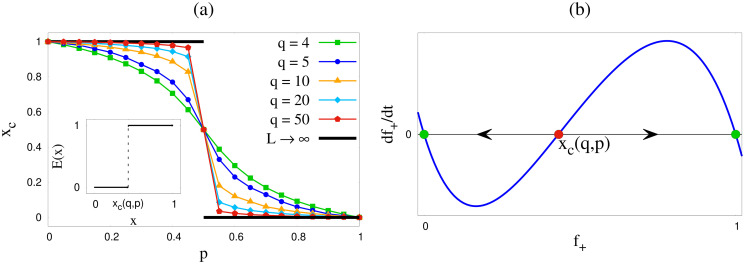
(a) Variation of *x*_*c*_ as a function of *p* for several values of *q* as obtained by mean field theory. The solid line in black denotes the results from Monte Carlo simulation for *L* → ∞. In large *q* limit, the mean field results converge to Monte Carlo results. *Inset* shows the behavior of exit probability as expected from the mean field estimations. (b) Generalised phase portrait for our model, where the green circles indicate stable fixed points at 0 & 1, and the red circle denotes the unstable fix point at *x*_*c*_. The values of *x*_*c*_ depend on *q* and *p*. The arrows indicate the directions of flow, such that for *f*_+_(*t* = 0) < *x*_*c*_, we would have *f*_+_(*t* → ∞) = 0 and similarly *f*_+_(*t* → ∞) = 1 for *f*_+_(*t* = 0) > *x*_*c*_.

This observation could however not be made from the simplified mean field results. If we define *x*_*c*_ as the cut-off value of initial fraction *x* of agents with positive opinion below which the system reaches a negative consensus, i.e., exit probability shows a step function (in the thermodynamic limit) at *x*_*c*_, then according to Monte Carlo results *x*_*c*_ = 1 for *p* < 0.5 and *x*_*c*_ = 0 for *p* > 0.5. However, the SMFT results show the existence of a non-trivial *x*_*c*_ for each *q*, as summarised in Fig 8(a). So *x*_*c*_ is basically an unstable fixed point, as shown in Fig 8(b). *x*_*c*_ was estimated by numerically solving Eq (14) and finding *x* below which the saturation value of *f*_+_ is 0. Although as *q* increases, analytically obtained values of *x*_*c*_ approaches that obtained by numerical simulation. This once again confirms that in the large *q* limit mean field results converge to Monte Carlo results.

Interestingly in the mean field theory, for any *q*, *x*_*c*_ = 0.5 for *p* = 0.5 (this is consistent with the discussions at the end of the subsection Mean field approach for any finite *q*, or for *q* → ∞). This implies that for *p* = 0.5, exit probability should show a step function at *x* = 0.5, according to mean field theory. However for infinite *q*, all points are fixed points for *p* = 0.5 which will give a linear exit probability. In the Monte Carlo simulation, as shown in Figs 4 and 5, exit probability indeed shows a linear behavior with *x* at *p* = 0.5 for several values of *q* ≤ 50 and also for several system sizes. A minor deviation from *p* = 0.5 would change this linear behavior to a step function like behavior in the thermodynamic limit as shown in Fig 6, which then agrees with mean field result in the large *q* limit.

### Dynamics

#### Relaxation behavior

The Monte Carlo simulations establish that the *p* = 0.5 point separates the two regions of consensus with positive opinion (for *p* > 0.5) and negative opinion (for *p* < 0.5) and that there is no other fixed point for any *q* (except for *p* = 1/2). One can also use symmetry arguments here as replacing simultaneously *p* by 1 − *p* and *f*_+_ by *f*_−_, the dynamics remain the same. However, how the system evolves towards these fixed points from arbitrary initial configurations is an important issue. Also there could be some associated time scale. In case of an exponential growth/decay of the relevant quantities, it is possible to define such a time scale (note that this is different from the exact time to reach the fixed point).

We found that the qualitative behavior of exit probability as well as the value of the exponent *ν* are independent of the exact value of *q*. Here, we report how the dynamics are affected by the value of *q*. Eq (7) shows that when *q* = 2, for any initial value, at large but finite times, the behavior of *f*_+_(*t*) is either 1 − *αe*^−*βt*^ or *α*′*e*^−*β*′*t*^ for *p* < 0.5 with *β* = 2*p* − 1.

We conjecture from this that for any *q*, *f*_+_ will have the form

$$\begin{array}{cc} f_{+}\left(t\right) & =\alpha e^{-\beta t},\;\text{for}\;p<0.5\\ & =1-\alpha^{\prime }e^{-\beta^{\prime }t},\;\text{for}\;p>0.5. \end{array}$$ (22)

In general *β* and *β*′, functions of *p*, can be different as found for *q* = 3 (Eqs 12 and 13). The coefficients *α*, *α*′ are trivially related to the initial values.

**Fig 9:**
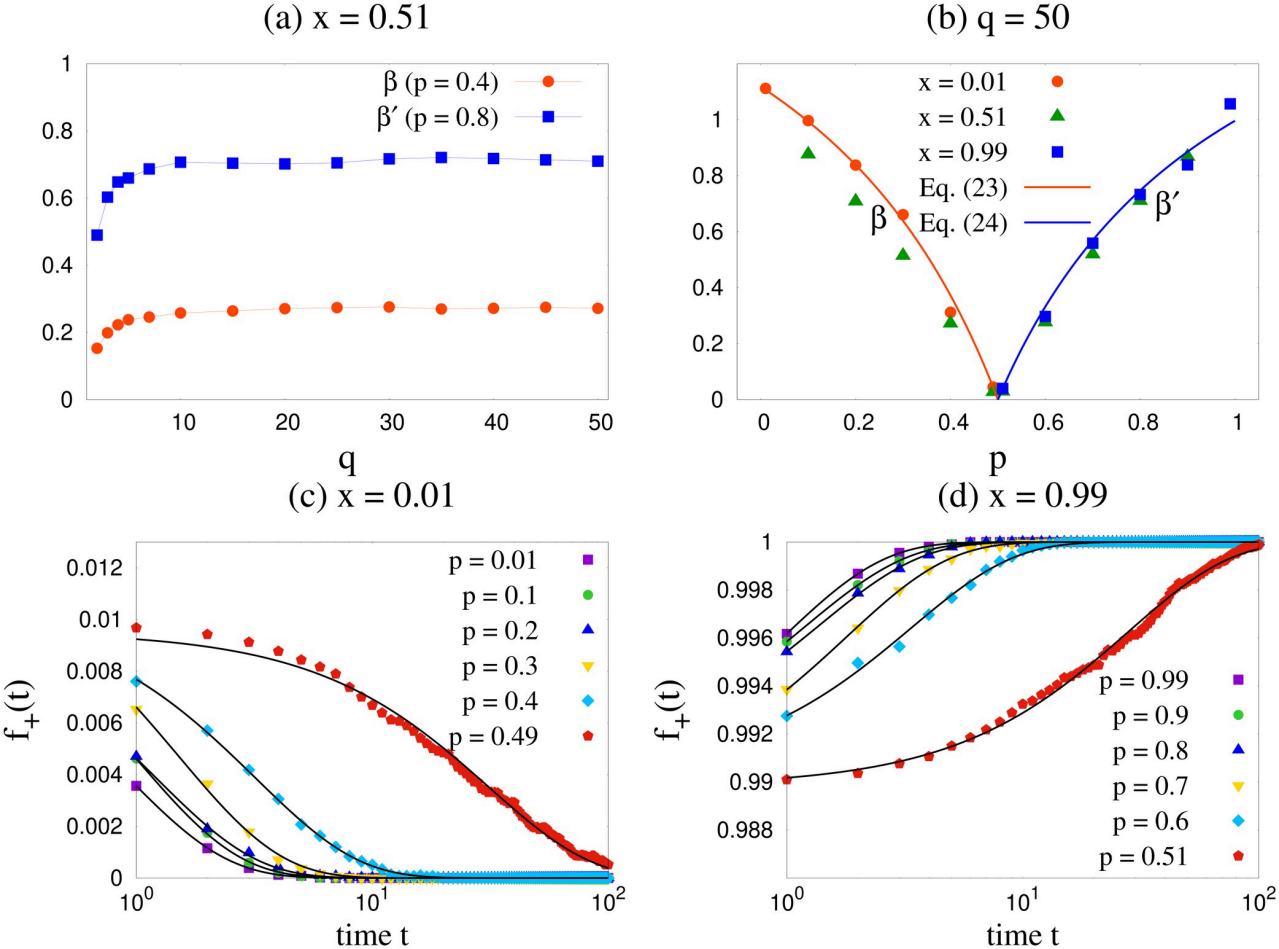
Exponential fittings of *f*_+_(*t*) curves. (a) shows the obtained values of *β* and *β*′ as a function of *q* for 2 typical values of *p* keeping *x* fixed. In (b) we show the obtained values of *β* and *β*′ as a function of *p*. The data is fitted according to Eqs (23) and (24). (c) and (d) shows the fitting of the *f*_+_(*t*) curves for *q* = 50 according to Eq (22) for 2 typical values of *x*, viz. *x* = 0.01 in the region *p* < 0.5 and *x* = 0.99 in the region *p* > 0.5 respectively.

In Fig 3 we fit the time dependence of *f*_+_ in the above form for two typical values of *p*, and for several values of *q* from 2 to 50. The results for *β*, *β*′ are different for the two values of *p* as expected but become independent of *q* for large values of *q*, as shown in Fig 9a. For small *q* values there is an increase with *q*.

In order to obtain the dependence of *β*, *β*′ on *p* from the SMFT for large *q*, we take note from Eqs (19) and (20) the variation of *δ* (which is linearly related to *f*_+_) with *p*. We argue that since *δ* cannot increase indefinitely, it is advisable to extract the values of the parameters from their vanishing feature. For the region *p* < 0.5, *δ* goes to zero in Eq (18) and for *p* > 0.5, *δ* goes to zero in Eq (17). Now the expressions for $f_{+}=f_{+}^{*}+\delta$ with $f_{+}^{*}=0,1$ are in the form of Eq 22 with the values of *β*, *β*′ given by

$$\begin{array}{c} \beta (p)∼\frac{1-2p}{1-p}\;\text{for}\;p<0.5 \end{array}$$ (23)

$$\begin{array}{c} \beta^{\prime }\left(p\right)∼\frac{2p-1}{p}\;\text{for}\;p>0.5 \end{array}$$ (24)

In Fig 9c and 9d, the results for *f*_+_ for a large value of *q* = 50 and different *p* values are shown from which the numerical estimates of *β*, *β*′ are made. The comparison with the theoretical estimates shows very good consistency. To extract the values of the parameters and compare them with the theoretical ones, we used *x* = 0.01 and *x* = 0.99 as the initial values of *f*_+_ in the two regions *p* < 0.5 and *p* > 0.5 respectively as the linear stability analysis is valid for small *δ* deviating from the fixed points zero and 1. We have found that for other values of *x* also, the *p* dependence of *β*, *β*′ are similar, apart from some trivial multiplicative factors shown in Fig 9b. One can conclude from this that there is a timescale which diverges as *p* → 0.5 from either side. Both the timescales are inversely proportional to *β*, *β*′ and therefore ∝ (2*p* − 1)^−1^. The point *p* = 0.5 can therefore be interpreted as a dynamical critical point manifesting critical slowing down.

#### Consensus times: Dependence on system size

**Fig 10:**
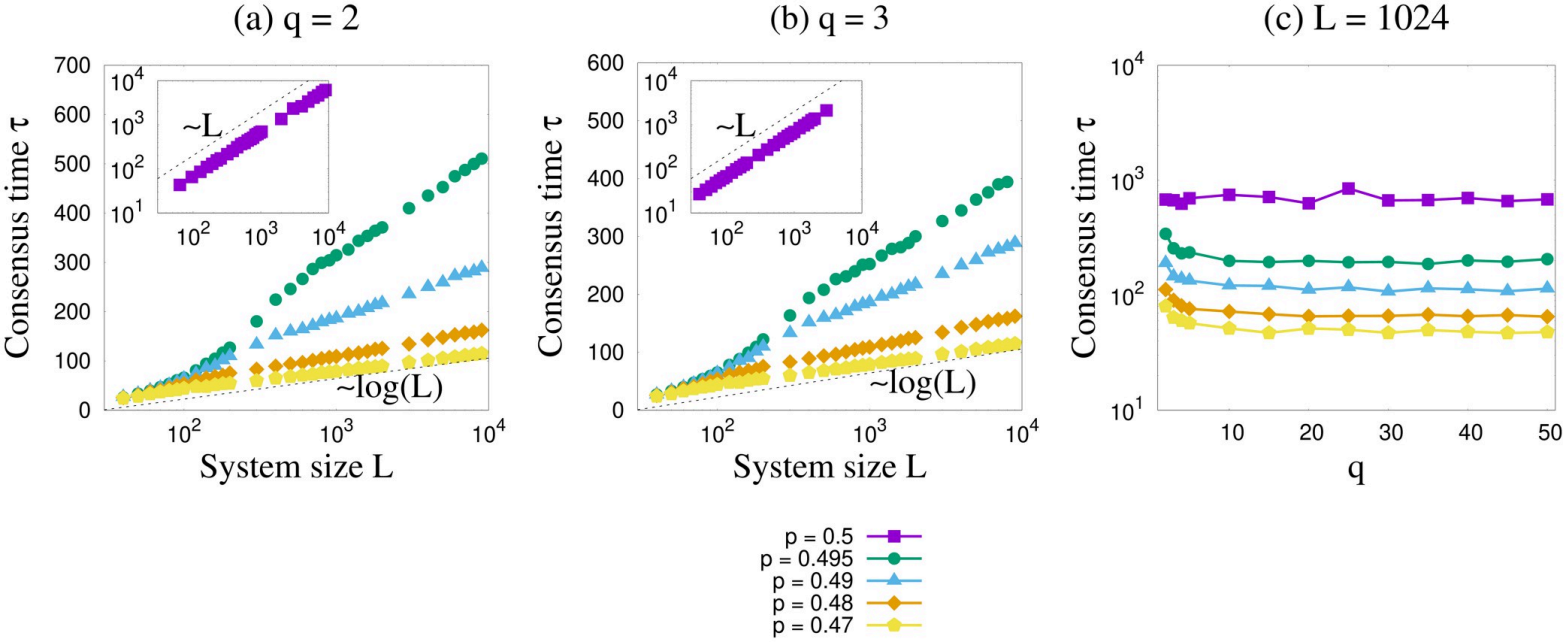
Consensus time *τ* as a function of system size *L*. Plots are shown for several values of *p* for (a) *q* = 2 and (b) *q* = 3. Insets show the data for *p* = 0.5. The simulations were done for *x* = 0.5. We can see that *τ* ∼ *L* for *p* = 0.5, but as *p* deviates from 0.5 the variation takes a logarithmic form. In (c) we show the variation of *τ* as function of *q* for system size *L* = 1024. *τ* decreases for lower values of *q*, however it does not exhibit a systematic dependence as *q* is made larger.

From the Monte Carlo simulations, one can estimate the time to reach the consensus states as a function of the system size. For the unbiased case, the dependence is a linear relation while for *p* ≠ 0.5 the results indicate a logarithmic variation, as also shown in [27]. The linear relation is also found in the mean field voter model. The results clearly show that the dynamics are much faster for any value of *p* different from 0.5. Fig 10 shows that the variation of the times are nearly independent of *q* as *q* is made larger consistent with the other results obtained.

## Conclusion

In this paper, we have studied a dynamical model of opinion formation where the opinion of an individual is determined on the basis of the opinions of other *q* number of agents. These *q* agents are basically the social connections with which the individual has an interaction. A parameter *p* determines the influential power of the agents with positive opinion. So *p* acts as a bias in the system; for *p* = 0.5, the model is identical to a mean field voter model. We analysed the dynamics of the system in terms of the fraction of agents with positive/negative opinion and also the steady states.

We obtain three regions, *p* < 0.5, *p* = 0.5 and *p* > 0.5 that determine the fate of the system. Interestingly, the equilibrium results for the ensemble averaged quantities, are independent of *q* in the thermodynamic limit. It implies that the size of the social connections influencing an agent is irrelevant and only the bias *p* matters as found from the simulation results. However, at small *q* values, the results related to the dynamic behavior are quantitatively *q* dependent, for example, the relaxation timescales and consensus times show a weak dependence on for *q* < 10 approximately. We argue that as *q* increases, the fluctuations in the opinions in the *q*-panel becomes less effective and as a result one gets *q* independent behavior for large *q*. This is analogous to mean field theory being valid at higher dimensions in general.

The mean field results for *q* = 2, 3, derived with all possible composition of the *q*-panel show very good agreement with the simulation results. In these cases, as well as in the simulations, there are only two fixed points for *p* ≠ 0.5. On the other hand, the simplified mean field rate equations derived for higher values of *q* are formulated assuming an average number of *n* = *qf*_+_ agents with opinion + 1 in the *q*-panel. So for all the cases where there is no unanimity, a single configuration is considered with this value of *n*. This assumption implies that the distribution of opinions in the *q*-panel, which is a subset of the whole system, is taken to be identical to the bulk and fluctuations are ignored. Comparison with numerical simulations indicates that the existence of the third fixed point in the SMFT results from this assumption. However, we found by numerically solving the equations that this third fixed point is an unstable one so except for the case when one starts from exactly at this fixed point, the final states are consensus states with either all positive/all negative opinions. If one starts with a value of *f*_+_ above (below) this fixed point, the all positive (negative) consensus state is reached. Therefore the step function for exit probability, according to SMFT, occurs at a *p*, *q* dependent value. Of course for *q* very large, the simplified mean field theory shows the existence of only two fixed points; this happens as neglecting fluctuations in the *q*-panel does not affect the results anymore. Although SMFT results show a ‘fictitious’ third (unstable) fixed point, it can be useful: SMFT maybe considered to correspond to an independent model where *n* is approximated by *qf*_+_ when the *q* panel is not unanimous.

The outcome of the study is quite simple but not obvious: even an infinitesimal initial bias towards the positive opinion will lead to a consensus state with all agents having positive opinion when the bias *p* is greater than 0.5. For *p* < 0.5, similarly, the all negative consensus state is reached for an initially biased (however small) state towards negative opinions. So the effect of minority spreading can occur here strongly and these results are independent of *q* in the thermodynamic limit.

The exponent *ν* obtained from the data collapse of the exit probability is shown to be universal and very close to unity. This value is different from that found in [18] where there is no dependence on the composition of the *q*-panel. The exit probability behaves differently in this model as there are only two fixed points in the present model while in the previous versions of *q*-voter models, one can have more than two.

While the equilibrium features of the model are independent of *q*, the dynamical behavior do show *q* dependence, at least for small *q*. We have made a linear stability analysis for *q* = 2, 3 and *q* → ∞ to show that the corresponding dynamical behavior are different. However, again we find that as *q* is made larger, the exponents are independent of *q*. One interesting observation is that one can identify *p* = 0.5, the mean field voter model point, as a dynamical critical point as critical slowing down occurs close to it. The corresponding timescale diverges with an universal exponent equal to unity according to the mean field theory and supported by numerical estimates. The system size dependence of the consensus time *τ* is again identical for all *q* and shows a logarithmic dependence for *p* ≠ 0.5. For *p* = 0.5, it is linear. Hence this provides further proof that the system behaves very differently as *p* deviates from 0.5.

In summary, we presented a model, where one of the opinion holds an “edge” making the agents with that opinion more influential. Out of the two parameters used, *p* happens to determine the qualitative behavior entirely. Quantitative results become *q* independent as *q* is made larger. The findings of this study illustrate how minimal initial biases can drive significant collective outcomes. This reflects real-world scenarios where small but consistent influences—such as targeted advertising, political messaging, or leadership strategies—shape group decisions. The model’s capacity to capture the independence of long-term outcomes from group size provides a framework to understand phenomena such as the diffusion of innovation or tipping points in social movements. By connecting these results to practical domains, the study contributes to theoretical understanding and offers potential applications in sociological research, marketing analytics, and strategic decision-making.

## Supporting information

S1 File Codes used for Monte Carlo simulations. This is a zip file which contains all the code files that were used for estimating *f*_+_, *E*(*x*) and *τ* in our model.
(ZIP)
